# From the diagnostic investigations of goiter to the diagnosis of lung cancer – case study

**DOI:** 10.1186/1756-6614-7-1

**Published:** 2014-02-01

**Authors:** Monika Koziołek, Anna Sieradzka, Lilianna Osowicz-Korolonek, Ewa Wentland-Kotwicka, Katarzyna Karpińska-Kaczmarczyk, Anhelli Syrenicz

**Affiliations:** 1 Department of Endocrinology, Metabolic Diseases and Internal Diseases, Pomeranian Medical University in Szczecin, Szczecin, Poland; 2 Clinical Specialized Ambulatory of Endocrinology, Autonomous Public Clinical Hospital No. 1 of Pomeranian Medical University in Szczecin, Szczecin, Poland; 3 Department of Patomorphology, Pomeranian Medical University in Szczecin, Szczecin, Poland

**Keywords:** Thyroid metastases, Lung cancer, Histopathology, Immunohistochemistry

## Abstract

Thyroid metastases account for approximately 1.4-3% of all malignancies of the thyroid gland. Thyroid metastases are most common in: clarocellular carcinoma of the kidney, lung cancer, breast cancer, malignant melanoma and cancers of gastrointestinal tract. A rare situation is when thyroid metastasis is diagnosed before detecting primary malignant focus and when it is the first manifestation of underlying disease. We present a case of 64-year-old male with thyroid metastasis being the first manifestation of lung adenocarcinoma.

The authors emphasize that patients with the history of malignancy should undergo an ultrasound examination of the thyroid gland in order to exclude a focal lesion, and if such lesion is detected, fine-needle aspiration biopsy is recommended. The authors also point out that establishing final diagnosis of thyroid metastasis of cancer in other organs is only possible on the basis of postoperative histopathology and immunohistochemistry.

## Background

Goiter is the most common pathology of the thyroid gland. Palpable focal lesions of the thyroid are found in 3-7% adults. Autopsy showed goiter in 50% of clinically normal thyroid glands. The rate of cancer in multinodular goiter is estimated at approximately 5% [1]. Thyroid cancer is the most common malignancy of endocrine system. Its most frequent histologic type is papillary cancer followed by follicular cancer, accounting for approx. 80% and approximately 10% of all thyroid cancers, respectively [2]. The thyroid gland may be the target for metastases. Thyroid metastases make up approx. 1.4-3% of all malignancies of the thyroid gland [3]. According to autopsy results, the rate of thyroid metastases may be as much as 8-times higher, and it is estimated even at 24% in patients with generalized malignant disease [3,4]. This difference between clinical reports and autopsy findings is related to clinically mute micrometastases. Thyroid metastases are most common in clarocellular carcinoma of the kidney, lung cancer, breast cancer, cancers of gastrointestinal tract and malignant melanoma [3-6]. In most cases, the diagnosis of thyroid metastases is established in the course of diagnostic investigations and the assessment of newly diagnosed cancer advancement, or in patients with the history of malignancy. Rarely have thyroid metastases been diagnosed before detecting primary malignant focus and being its first manifestation [3,6]. We present a case of 64-year-old male in whom the diagnosis of focal lesion of the thyroid gland led to the detection of lung adenocarcinoma.

### Case presentation

A 64-year-old male patient with hypertension, ischemic heart disease, history of myocardial infarction in 1999 and lung tumors monitored in consecutive CT scans attended a visit at Endocrine Outpatient Clinic because of a palpable tumor in lower part of the neck on its right side. Anomalies found in physical examination included the enlargement of the right thyroid lobe with palpable hard tumor with the diameter of approx. 4 cm and without enlargement of cervical lymph nodes. Serum TSH (thyroid stimulating hormone) level was 3.0 uIU/ml (normal range 0.27-4.2 uIU/ml). An ultrasound examination of the thyroid gland showed a solid, hypoechogenic focal lesion with microcalcifications in the right thyroid lobe – lesion diameter was 40 mm [7]. Cytology of biopsy material sampled from thyroid tumor suggested follicular tumor. Immunocytochemistry proved that bioptate reaction to calcitonin was negative. The patient was qualified for surgery and in November 2009, right thyroidectomy was performed. After surgery, the patient required treatment with L-thyroxine at the dose of 37.5 ug/day. The removed right thyroid lobe - size 7 x 4 x 3 cm with the tumor diameter 3.5 cm – was subjected to histopathology assessment. The tumor was encapsulated by connective tissue and was made of cylindrical epithelial cells with large nucleoli and mitotic figures. Immunohistochemistry showed negative reaction to thyroglobulin, CK20, calcitonin and CD34, while reactions to CK7 and vimentin were positive. Based on histopathology and immunohistochemistry results, it was concluded that the thyroid tumor was a metastasis, not a primary thyroid cancer.

The search for primary malignant focus started with CT of abdomen with contrast enhancement (January 2010), which did not show any lesions in the liver, spleen, pancreas, adrenal glands, retroperitoneal space and small pelvis; the lymph nodes in the regions covered by CT scan were not enlarged, either. CT scan also covered the basal part of the lungs showing the progression of subpleural tumor in VII segment of right lung from 8 mm to 13 mm as compared to CT scan of September 2009. Consequently, the patient was referred to the Department of Lung Diseases. Fiberoptic bronchoscopy revealed a hypertrophic lesion in S4 segment of left lung, which occluded bronchial lumen. Histopathology revealed that the lesion was adenocarcinoma (*adenocarcinoma typus bronchioalveolaris).* The patient was qualified for chemotherapy. In 2011, the patient developed bone metastases and therefore, radiotherapy was started. In 2011, the patient attended regular follow-up visits at Endocrine Outpatient Clinic every 3 months, he felt well and despite advanced stage of the disease, his general condition was good. In January 2012, check-up ultrasound of the neck was performed showing three solid normoechogenic focal lesions in the left thyroid lobe – their sizes being 6 x 5 x 4 mm, 10 x 8 x 9 mm and 4 x 4 x 3 mm – and a fragment of thyroid parenchyma size 4 x 3 mm was found in the right lobe bed. Additionally, at mid-length of the neck, on the right side and close to internal carotid artery, there was a hypoechogenic, heterogeneous solid lesion, with blurred contours and the size of 15 x 10 mm, which looked suspicious in oncological terms. Fine-needle aspiration biopsy of the largest focal lesion from the left thyroid lobe revealed sheets of follicular cells without cytology features of malignancy, while the biopsy of the lesion at the right side showed non-small cell cancer cells. On his last visit at Endocrine Outpatient Clinic in March 2012, the patient’s general condition was good, and he remained in the state of clinical and biochemical euthyreosis. He continued to be treated by pulmonologist. According to the information obtained from patient’s family, the malignant disease progressed rapidly and the patient died in June 2012.

## Discussion

Thyroid metastasis may be the first manifestation of malignancy in another organ [3,6,8]. Lam et al. analyzed a group of 79 patients with thyroid metastases and found that in 3 patients with lung cancer and 1 with breast cancer, thyroid metastasis was the first clinical manifestation of malignancy [8]. In clinical terms, the symptoms of thyroid metastases do not differ from those of primary thyroid cancer. Neck circumference increases, thyroid gland gets harder or palpable thyroid tumors develop. As the disease progresses, the patient may feel: new or enlarging palpable thyroid nodule, pressure in the neck, hoarseness, stridor, cough or dysphagia and haemoptysis [3,6,9]. In the majority of patients with thyroid metastases, the function of the thyroid gland remains normal but in rare cases, both hypothyroidism and thyrotoxicosis have been described [3,9-12]. Miyakawa M. et al. were the first to describe thyrotoxicosis in female patient with thyroid metastasis of lung adenocarcinoma, which resulted in massive damage of thyroid follicular cells [10].

Thyroid metastases are rarely diagnosed based on cytology assessment, especially in patients without clinical features suggesting malignancy of another organ [5,6]. Histopathology and immunohistochemistry are decisive in the diagnosis of thyroid metastasis. In patients with postoperative diagnosis of thyroid metastasis based on cytology of fine-needle aspiration biopsy material, follicular neoplasm, neoplastic cells, atypical cell, as well as primary cancer of the thyroid gland were suspected during initial diagnosis of goiter [3,6,8,12]. Lam et al. were the first to describe two cases of thyroid metastases, which were diagnosed as primary thyroid cancer based on cytology assessment, while in other two cases described in this paper cytology results suggested thyroid metastases but these were patients with the history of malignancy [8]. Haraguchi et al. reported thyroid metastasis of lung adenocarcinoma, imitating primary cancer of the thyroid gland [13]. In a group of 15,122 patients analyzed by Buła G. et al. and operated for various forms of goiter, 10 patients were diagnosed with thyroid metastases based on histopathology result. Preoperative cytology assessment showed thyroid metastasis only in one of these patients. In the remaining subjects, cytology results included follicular tumor, atypical cells, neoplastic cells or epithelial cells and atypia [5].

The differentiation between primary thyroid cancer and thyroid metastasis of lung cancer is particularly difficult, because the thyroid gland and lungs have common embryogenesis and therefore, they have similar profile in immunohistochemical staining [14]. Immunohistochemical staining for TTF-1 (*thyroid transcription factor-1*), thyroglobulin, CEA (*carcinoembryonic antigen*), cytokeratins 7 and 20, vimentin and histochemical staining for mucus in cancer cells may be used in order to detect primary malignant focus [15,16]. The staining for CEA, thyroglobulin and calcitonin of both cytology material and tumor samples are the most useful in the differential diagnosis. Differentiated thyroid cancers exhibit positive reaction to thyroglobulin. Medullary thyroid cancer cells have positive reaction to calcitonin, CEA and chromogranin, while they do not contain thyroglobulin [17].

Lung adenocarcinoma may form glandular structures, which may resemble thyroid follicles, particularly in cytology; therefore, just as in our case, the final diagnosis of thyroid metastasis with indication of primary malignant focus is based on histopathology and detailed analysis of immunohistochemical profile of neoplastic cells.

## Conclusions

1. An ultrasound examination of the thyroid gland is recommended in patients with neoplastic disease and if focal lesion is found, fine-needle aspiration biopsy is necessary.

2. Thyroid metastasis may be the first clinical manifestation of neoplasm originating from another organ.

3. Suspecting follicular neoplasm in cytology material does not exclude the diagnosis of thyroid metastasis of other organ malignancy in postoperative histopathology.

4. The final diagnosis of thyroid metastasis with indication of primary malignant focus is based on histopathology and detailed analysis of immunohistochemical profile of neoplastic cells.

## Consent

Written informed consent was obtained from the patient's daughter for the publication of this report and any accompanying images. Patient died before the publication.

## Abbreviations

CT: Computed tomography; TSH: Thyroid stimulating hormone; CEA: Carcinoembryonic antygen; TTF-1: Thyroid transcription factor-1.

## Competing interests

The authors declare that they have no competing interests.

## Authors’ contributions

MK has analyzed the data, has investigated the subject based on various publications and conducted general supervision of the research group; AS has collected and analyzed the data, has investigated the subject based on various publications; LOK has investigated the subject based on various publications EWK has treated the patient, has collected the data and investigated the subject based on various publications, KKK has made histopatology and immunohistochemical profile, investigated the subject based on various publications SA has given final approval of the version to be published and revised it critically for important intellectual content. All authors read and approved the final manuscript.
